# Accelerated diffusion-weighted magnetic resonance imaging at 7 T: Joint reconstruction for shift-encoded navigator-based interleaved echo planar imaging (JETS-NAViEPI)

**DOI:** 10.1162/imag_a_00085

**Published:** 2024-02-05

**Authors:** Zhengguo Tan, Patrick Alexander Liebig, Robin Martin Heidemann, Frederik Bernd Laun, Florian Knoll

**Affiliations:** Department Artificial Intelligence in Biomedical Engineering (AIBE), Friedrich-Alexander University of Erlangen-Nuremberg, Erlangen, Germany; Siemens Healthcare GmbH, Erlangen, Germany; Institute of Radiology, University Hospital Erlangen, Friedrich-Alexander University of Erlangen-Nuremberg, Erlangen, Germany

**Keywords:** Diffusion-weighted magnetic resonance imaging, echo planar imaging, navigator, ultra-high field, joint reconstruction, low rank

## Abstract

The pursuit of high spatial-angular-temporal resolution for in vivo diffusion-weighted magnetic resonance imaging (DW-MRI) at ultra-high field strength (7 T and above) is important in understanding brain microstructure and function. Such pursuit, however, faces several technical challenges. First, increased off-resonance and shorter $T_{2}$ relaxation require faster echo train readouts. Second, existing high-resolution DW-MRI techniques usually employ in-plane fully-sampled multi-shot EPI, which not only prolongs the scan time but also induces a high specific absorption rate (SAR) at 7 T. To address these challenges, we develop in this work navigator-based interleaved EPI (NAViEPI) which enforces the same effective echo spacing (ESP) between the imaging and the navigator echo. First, NAViEPI renders no distortion mismatch between the two echoes, and thus simplifies shot-to-shot phase variation correction. Second, NAViEPI allows for a large number of shots (e.g., >4) with undersampled iEPI acquisition, thereby rendering clinically-feasible high-resolution sub-milliemeter protocols. To retain signal-to-noise ratio (SNR) and to reduce undersampling artifacts, we developed a $k_{y}$-shift encoding among diffusion encodings to explore complementary k- q-space sampling. Moreover, we developed a novel joint reconstruction with overlapping locally low-rank regularization generalized to the multi-band multi-shot acquisition at 7 T (dubbed JETS-NAViEPI). Our method was demonstrated, with experimental results covering 1 mm isotropic resolution multi b-value DWI and sub-millimeter in-plane resolution fast TRACE acquisition.

## 1 Introduction

Diffusion-weighted magnetic resonance imaging (DW-MRI) (Le Bihan et al., 1986; Merboldt et al., 1985) is a non-invasive modality that is sensitive to the intravoxel Brownian motion of water molecules. DW-MRI forms the basis for diffusion tensor imaging (DTI) (Basser et al., 1994; Mori et al., 1999) and high angular resolution diffusion imaging (HARDI) (Tuch et al., 2002), and has been widely used in acute brain ischemia diagnosis, in tumor detection and staging, and in neuroscience (Jones, 2010).

For DW-MRI acquisition, the commonly used pulse sequence is single-shot echo-planar imaging (SS-EPI) (Mansfield, 1977). SS-EPI is capable of rapidly acquiring one DW image per radio-frequency excitation at the order of 100 ms, and is thus motion robust. However, conventional SS-EPI, even with three-fold accelerated acquisition (Bammer et al., 2001) using parallel imaging (Griswold et al., 2002; Pruessmann et al., 1999; Ra & Rim, 1993; Roemer et al., 1990), still suffers from low spatial resolution and geometric distortions.

In the quest for high spatial-angular-temporal-resolution and minimal-geometry-distortion DW-MRI, tremendous efforts have been made. Techniques for the correction of image distortions induced by off-resonances and eddy currents have been developed (Andersson et al., 2003). Furthermore, gSlider (Setsompop et al., 2018) with blipped-CAIPI (Setsompop et al., 2012) for simultaneous multi-slice (SMS) (Breuer et al., 2005; Maudsley, 1980) was proposed to achieve high-resolution DW-MRI. Advanced pulse sequences based on multi-shot EPI have also been developed, including but not limited to interleaved EPI (iEPI) (Butts et al., 1993), PROPELLER (Pipe et al., 2002), and readout-segmented EPI (rsEPI) (Heidemann et al., 2010; Porter & Heidemann, 2009).

Based on four-shot iEPI, multiplexed sensitivity encoding (MUSE) image reconstruction achieved DW-MRI with a sub-millimeter in-plane resolution and maximal b-value 800 s/mm^2^ at 3 T (Chen et al., 2013). The four-shot iEPI employed in MUSE acquired an in-plane fully-sampled k-space, except partial Fourier. Every shot (segment), corresponding to four-fold undersampling, was then reconstructed via parallel imaging to obtain shot-to-shot phase variation. This indicates that increasing the number of shots in MUSE will result in higher undersampling per shot, and consequently, degrade shot phase estimation (Wu & Miller, 2017).

Alternatively, navigator-based iEPI acquisition has been proposed (Dai et al., 2017, 2018; Jeong et al., 2013). These proposals allow for a larger number of shots, and hence higher spatial resolution. However, due to the use of different ESP between the imaging echo and the navigator echo, these proposals suffered from geometric distortion mismatch between the two echoes and thus required specific compensation methods. In contrast, rsEPI (Heidemann et al., 2010; Porter & Heidemann, 2009) used the same readout segment for both echoes, and thus required no distortion correction of navigator echoes.

Beyond the MUSE-type parallel imaging reconstruction, compressed sensing (Block et al., 2007; Lustig et al., 2007) has been explored. For instance, multi-shot reconstruction techniques based on structured low-rank matrix completion (MUSSELS) (Bilgic et al., 2019; Mani et al., 2017) achieved five-shot DW-MRI with nine-fold undersampling per shot. Recently, JULEP (Dai et al., 2023) incorporated explicit phase mapping into MUSSELS. These reconstruction techniques, that is, MUSE, MUSSELS, and JULEP, targeted the reconstruction of one DW image from interleaved EPI acquisition, and did not explore joint- k- q-space undersampling or reconstruction.

Joint- k- q-space undersampling can be achieved via proper regularization along the diffusion encoding direction. Relevant examples are diffusion undersampling with Gaussian process estimated reconstruction (DAGER) (Wu et al., 2019) and magnitude-based spatial-angular locally low-rank regularization (SPA-LLR) (Hu et al., 2020). However, DAGER addressed the reconstruction problem of single-shot EPI acquisition and SPA-LLR focused on the reconstruction of single-band and fully-sampled iEPI acquisition.

In this work, we propose a Joint k- q-slice rEconsTruction framework for Shift-encoded NAVigator-based interleaved EPI at 7 T (dubbed JETS-NAViEPI). Our pulse sequence, NAViEPI, differs from most existing techniques. First, NAViEPI builds upon interleaved EPI, thereby allowing for fast and efficient k-space coverage. Second, inspired by rsEPI, NAViEPI ensures the same effective ESP between the imaging and the navigator echo, thereby minimizing geometric distortion and allowing for the use of a larger number of shots. NAViEPI essentially integrates the advantages of both iEPI and rsEPI. Third, NAViEPI utilizes undersampled multi-shot iEPI, thereby alleviating the SAR problem at 7T. Fourth, NAViEPI shifts the k-space in-plane sampling pattern along the phase encoding ($k_{y}$) direction. This shifting creates complementary k- q-space sampling, which leads to the possibility of our joint k- q-slice reconstruction. Specifically, we employ spatial-diffusion overlapping LLR regularization to jointly reconstruct all diffusion encodings and multi-band slices. In vivo experiments at 7 T and comparisons with other techniques demonstrate the efficiency of our proposed method in achieving high spatial resolution DW-MRI at ultra-high field.

## 2 Materials and Methods

### 2.1 Multi-band shift-encoded iEPI acquisition

**Fig. 1.**
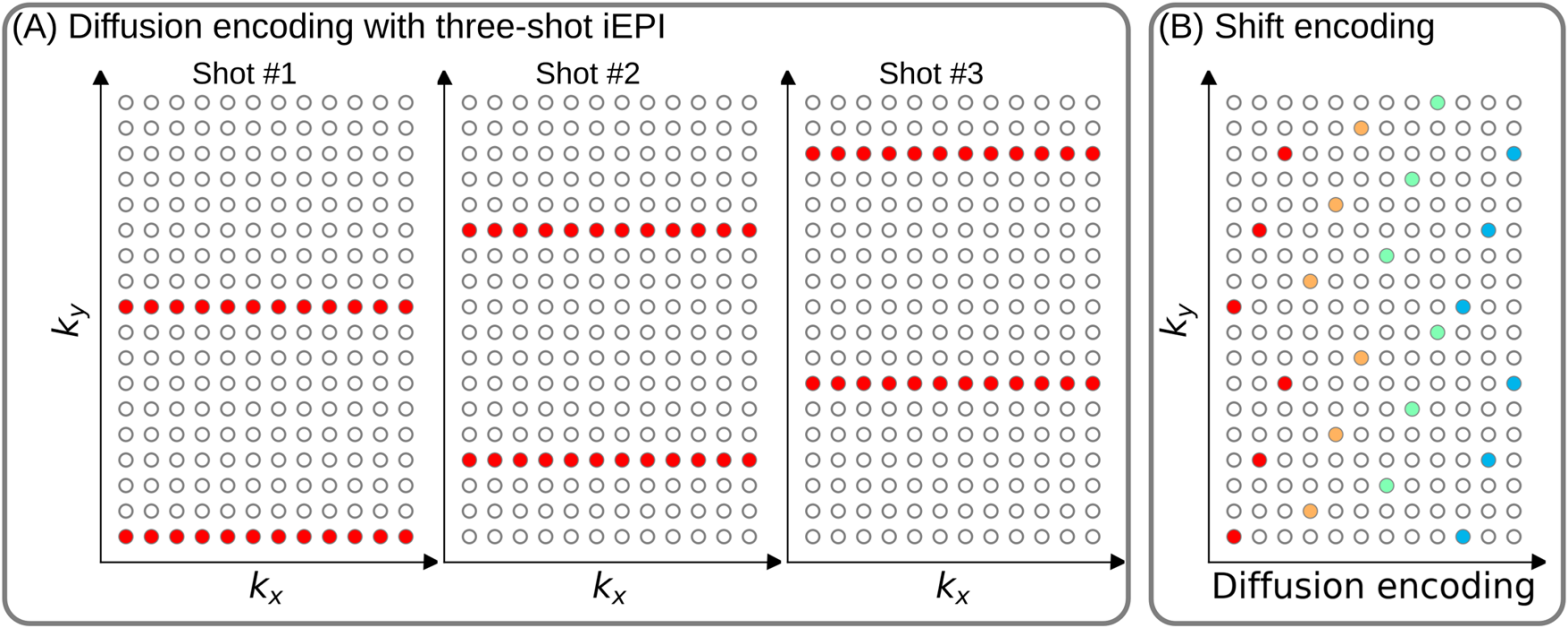
(A) An example DW-MRI acquisition with three-shot interleaved EPI acquisition. (B) The proposed $k_{y}$ shifted diffusion encoding scheme. This example employs three shots per DW image. Therefore, every three columns have the same color.

Figure 1 (A) displays the diffusion-weighted image acquisition based on three-shot interleaved EPI with three-fold in-plane undersampling. Conventionally, such a sampling pattern is repeated for all diffusion directions. In contrast, we propose the $k_{y}$-shifted diffusion encoding, as shown in Figure 1 (B). The interleaved EPI sampling pattern is shifted by one $k_{y}$ line per diffusion direction, with the cycling period being the in-plane undersampling factor.

It is worth noting that, as shown in Figure 1 (A), the undersampling factor of one segment is $R_{\text{in}-\text{plane}}\times N_{\text{shot}}$ (ignore multi-band undersampling here), yielding nine-fold in-plane undersampling in this example. In other words, the undersampling factor per segment linearly scales up with the number of shots. Consequently, conventional self-gating reconstruction techniques, for example MUSE, suffer from degraded shot-to-shot phase estimation, which, in turn, limits the number of shots and spatial resolution.

### 2.2 NAViEPI: navigator-based iEPI with consistent effective ESP between the imaging and the navigator echo—where iEPI meets rsEPI

**Fig. 2.**
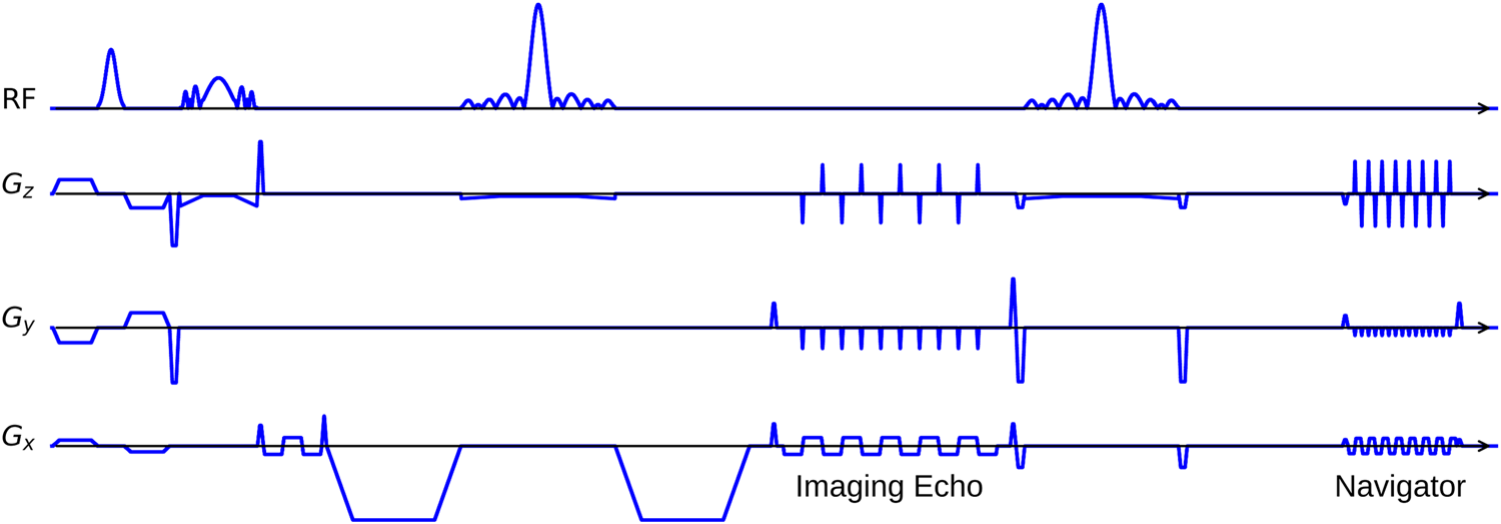
The NAViEPI sequence diagram. SMS is utilized for the acquisition of both imaging and navigator echoes. While the acceleration factor per navigator is the same as listed in Table 1, the acceleration factor per imaging echo is in addition linearly scaled by the number of shots.

Instead of the self-gated MUSE with in-plane fully-sampled iEPI and a limited number of shots, we propose NAVigator-based interleaved EPI (NAViEPI), as illustrated in Figure 2. Inspired by rsEPI (Porter & Heidemann, 2009), NAViEPI enforces a consistent effective ESP between the imaging and the navigator echo, thereby minimizing distortion mismatch between the two echoes.

**Table 1.**
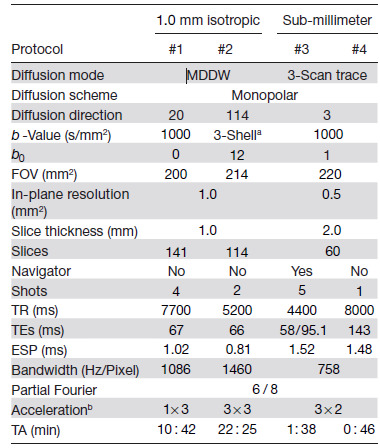
NAViEPI acquisition protocols.

a 3-Shell: 20, 30, and 64 directions with b-values of 1000, 2000, and 3000 s/mm^2^, respectively.

b Acceleration: Both in-plane and slice undersampling can be employed, denoted as ($R_{\text{in}-\text{plane}}\times R_{slice}$).

MDDW: multi-direction diffusion weighting; TA: total acquisition time.

Since one imaging echo presents one segment in multi-shot EPI acquisition, its effective ESP is defined as

$${\text{ESP}}_{\text{eff}}=\frac{\text{ESP}}{R_{\text{in}-\text{plane }}\times \;N_{\text{shot}}}$$ (1)

Here, a larger number of shots (segments) increases the undersampling factor per segment (see Fig. 1), but decreases the effective ESP. Since the navigator echo is acquired for each segment, its in-plane undersampling factor equals $R_{\text{in}-\text{plane}}$. Therefore, the effective ESP of the navigator echo must match that of the imaging echo, as given in Eq. (1). With a matching effective ESP, the base resolution of the navigator echo can then be determined.

### 2.3 In vivo acquisition protocols

We implemented multiple in-vivo acquisition protocols at a clinical 7 T MR system (MAGNETOM Terra, Siemens Healthineers, Erlangen, Germany) equipped with a 32-channel head coil (Nova Medical, Wilmington, MA, USA) and the XR-gradient system (maximum gradient strength 80 mT/m with a peak slew rate of 200 T/m/s). To calibrate coil sensitivity maps, reference scans employed a gradient-echo (GRE) sequence. Spectral fat saturation and mono-polar diffusion-encoding gradients were used. The phase-encoding direction was selected as anterior-to-posterior.

This study was approved by the local ethics committee. Three volunteers with informed consent obtained before scanning participated in this study. Detailed acquisition protocols are listed in Table 1. In the spirit of reproducible research, another volunteer with informed consent was recruited for the scan of all acquisition protocols, and the results were displayed in Supplementary Information (SI).

#### 2.3.1 20-Diffusion-direction acquisition at 1 mm isotropic resolution

As listed in Table 1, Protocol #1 with four-shot iEPI and without in-plane undersampling was implemented. This protocol represents the acquisition scheme employed in many existing multi-shot reconstruction techniques, (e.g., MUSE, SPA-LLR, and JULEP). The acquired data from this protocol served as ground truth. Different reconstruction methods, specifically JETS, MUSE, and JULEP, were compared. We compared with JULEP instead of MUSSELS, because JULEP uses not only structured low-rank constraints but also explicit phase mapping.

We then retrospectively reduced the four-shot data to only one shot per diffusion encoding without and with the proposed $k_{y}$ shifting to simulate four-fold in-plane undersampling. JETS reconstruction was performed on the fully-sampled data and the retrospectively undersampled data to validate the proposed $k_{y}$-shifted acquisition.

#### 2.3.2 Three-shell acquisition at 1 mm isotropic resolution

Protocol #2 in Table 1 was implemented for multi-shell diffusion tensor imaging (DTI) (Basser et al., 1994). We acquired a total of 114 diffusion directions, whereas $b_{0}$ measurements were interspersed every 10 diffusion directions. This protocol was used to demonstrate the capability of JETS in achieving high spatial-angular-temporal resolution.

#### 2.3.3 3-Scan trace acquisition at $0.5\times 0.5\times 2.0mm^{3}$ voxel size

As listed in Table 1, Protocol #3 was implemented based on NAViEPI with five shots per diffusion encoding. This protocol was compared against single-shot EPI (Protocol #4) with the same spatial resolution and acceleration, such as to demonstrate the sampling efficiency of NAViEPI.

### 2.4 Forward modeling

Our proposed acquisition method yields multi-dimensional multi-band k-space data $y_{c,q,s}$, where c, q, s denotes the index of the coil sensitivity map, the diffusion encoding, and the shot, respectively. Acquisition modeling needs to consider several aspects.

First, the acquired k-space data y is mapped from individual shot images $x_{q,s,z}$ via the forward model,

$$y_{c,q,s}=P_{q,s}\Sigma \Theta_{z}FS_{c}x_{q,s,z}$$

$$y:=E_{1}x$$ (2)

Here, the encoding matrix $E_{1}$ comprises a chain of linear operators. Every shot image x is point-wise multiplied by a set of coil sensitivity maps (S) and Fourier transformed (F). The output is then point-wise multiplied by the multi-slice phase map (Θ) with z the slice index in simultaneously excited slices. This operator shifts individual slice along the phase-encoding direction via varying phase modulation (Breuer et al., 2005). The SMS k-space data is then summed (collapsed, Σ) along the slice dimension and masked (point-wise multiplied, P) by the sampling pattern of each diffusion encoding and shot.

Second, for diffusion MRI based on multi-shot EPI, multiple shots acquired for a given diffusion encoding need to be combined as one DW image ($\tilde{x}$). One possibility is to perform magnitude average (Chen et al., 2013) or root-sum-squares (RSS) (Mani et al., 2017) of shot images. This method is robust to in-plane motion, but sub-optimal concerning SNR (Guhaniyogi et al., 2016). Alternatively, shot combination can be done via shot-to-shot phase variation correction (Chen et al., 2013; Liu et al., 2005). This can be incorporated into our formulation as point-wise multiplication between the shot-to-shot phase variation (Φ) and the DW image ($\tilde{x}$),

$$x_{q,s,z}=\Phi_{q,s,z}{\tilde{x}}_{q,z}$$ (3)

Note that $\tilde{x}$ can be obtained by applying the adjoint of Φ to x. In MUSE, Φ is obtained by parallel imaging reconstruction of all shots with subsequent phase smoothing of every shot image. Based on this phase correction, the complete forward model follows

$$y:=E_{2}\tilde{x}=E_{1}\Phi \tilde{x}$$ (4)

where the encoding matrix $E_{2}$ comprises the chain of the shot-to-shot phase variation Φ and the encoding matrix $E_{1}$. We implemented these two encoding operators in SigPy (Ong & Lustig, 2019).

### 2.5 Joint k- q-slice reconstruction

Based on the generalized forward models in Eqs. (2) and (4), our proposed joint k- q-slice reconstruction can be formulated as a three-step approach.

**I. Navigator echo reconstruction**. The acquisition of navigator echoes follows the forward model in Eq. (2), so the reconstruction of navigator echoes can be formulated as:

$${\text{argmin}}_{x}\left||y-E_{1}\;x\;\right|{\text{|}}_{2}^{2}\;+\lambda R\left(x\right)$$ (5)

where R(x) denotes the regularization functional with the regularization strength λ. In this work, $\ell^{2}$ regularization was used, that is, $R\left(x\right)=|\text{|}x\text{|}{|}_{2}^{2}$. In the case of self-navigating (i.e., no navigator acquired) as Protocol #2, the central k-space region (i.e., 1/4 of the full image matrix) of each segment is used as y in Eq. (5).

**II. Phase smoothing**. Shot-to-shot phase variation was extracted from the reconstructed navigator echo phases. Assuming that phase images are spatially smooth (Chen et al., 2013; Dai et al., 2023), we employed the adaptive Hanning filter to smooth shot phases,

$$x=F^{-1}{ℋ}^{K}Fx$$ (6)

where x is the reconstructed navigator image from Step I. ℋ is the Hanning window with the non-negative integer K. K controls the width of the Hanning window.

**III. Shot-combined reconstruction**. Joint reconstruction of all DW images using the shot-combined forward model $E_{2}$ with shot-to-shot phase variation from Step II reads:

$$\arg\min_{\tilde{x}}||y-E_{2}\tilde{x}|{|}_{2}^{2}+\lambda ||T(\tilde{x})|{|}_{*}$$ (7)

Here, LLR regularization was employed in the local spatial-diffusion matrices, based on the theory of partially separable functions (Liang, 2007; Trzasko & Manduca, 2011; Zhang et al., 2015). T represents a linear operator that firstly slides a local patch window through all DW images and then flattens every set of local patches to construct two-dimensional (2D) spatial-diffusion matrices. The spatial dimension equals the block size, and the diffusion dimension is the number of diffusion encodings. ${\left\|\;T\left(\tilde{x}\right)\;\right\|}_{\text{*}}$ is the nuclear norm, that is, the sum of singular values of a spatial-diffusion matrix. This nuclear norm regularization was accomplished via singular value thresholding (SVT) of each spatial-diffusion matrix (Cai et al., 2010). After SVT, the adjoint of T, $T^{H}$, was needed to reorder pixel values from the spatial-diffusion matrices back to DW images.

To alleviate checkerboard artifacts induced by LLR regularization with non-overlapping blocks (Hu et al., 2020), we employed overlapping blocks. In this case, values from overlapping positions are summed up to the output of $T^{H}$. To enable the correct use of $T^{H}$, we element-wise divided the output of $T^{H}$ by a scaling matrix. This matrix was obtained via $T^{H}(T(1))$, where 1 denotes the matrix of all ones with the same shape as the input x.

As the local patch window varies depending on the number of diffusion encodings or user selection, we implemented a singular-value spectrum normalization strategy to reduce the effect of the local patch window variation on regularization strength. Specifically, the singular values of constructed spatial-diffusion matrices were divided by the patch window width. After SVT, the thresholded singular values were multiplied with the patch window width for rescaling.

### 2.6 Reconstruction

The acquired raw data were read in by twixtools (https://github.com/pehses/twixtools). Ramp-sampling regridding and FOV/2-ghost correction were also performed in twixtools. Subsequently, coil sensitivity maps were computed from reference scans using ESPIRiT (Uecker et al., 2014) in SigPy (Ong & Lustig, 2019).

With this pre-processing as well as the implemented forward models and proximal operator, the inverse problem in Eq. (7) was solved by the alternating direction method of multipliers (ADMM) (Boyd et al., 2010).

ADMM solves the minimization problems in an alternating update scheme,

$$\left\{\begin{array}{l} X^{\left(k+1\right)}\;\;\;:=\arg\min||y-E(x)|{|}^{2}+\rho /2||Tx-z^{\left(k\right)}+u^{\left(k\right)}|{|}_{2}^{2}\\ z^{\left(k+1\right)}\;\;\;\;\;:=T_{\lambda /\rho }(Tx^{\left(k+1\right)}+u^{\left(k\right)}\\ u^{\left(k+1\right)}\;\;\;\;:=u^{\left(k\right)}+Tx^{\left(k+1\right)}-z^{\left(k+1\right)} \end{array}\right.$$ (8)

where k denotes the ADMM iteration, z is the auxiliary variable (z=Tx), and u is the Lagrangian multipliers. Importantly, when solving Eq. (2), x denotes shot images and E denotes $E_{1}$ in Eq. (8). In contrast, x denotes shot-combined images and E denotes $E_{2}$ when solving Eq. (4). x can be solved using linear least square algorithms, for example, conjugate gradients (Hestenes & Stiefel, 1952), while z is updated via singular value thresholding (T) with the thresholding parameter λ/ρ. The coupling parameter ρ is effective in both the update of x and z. It acts as Tikhonov regularization strength when updating x, but also inversely scales the thresholding strength when updating z.

In this work, 15 ADMM iterations with ρ=0.05 and λ=0.01 were used. All reconstructions were done on a single A100 SXM4/NVLink GPU with 40GB memory (NVIDIA, Santa Clara, CA, USA).

We compared our proposed joint reconstruction with established multi-shot reconstruction techniques, specifically, MUSE (Chen et al., 2013) and JULEP (Dai et al., 2023), hosted on GitHub by Dr. Dai (Dai et al., 2023). Further, we performed the local-PCA denoising (Cordero-Grande et al., 2019) as implemented in MRtrix (Tournier et al., 2019) on the MUSE reconstructed complex DW images.

The in vivo data acquired from Protocol #2 in Table 1 consisted of 126 diffusion directions, which exceeds the available GPU memory. Therefore, the 126 data volumes were uniformly split into three parts for our JETS reconstruction with an LLR block width of 6 and the LLR regularization in both Steps I and III in Section 2.5. In addition, MUSE reconstruction was also performed, followed by the local-PCA denoising. Reconstructed DWIs were then processed by DiPy (Garyfallidis et al., 2014) to obtain color-coded fractional anisotropy (cFA) maps.

## 3 Results

### 3.1 Smoothing of shot-to-shot phase variation

**Fig. 3.**
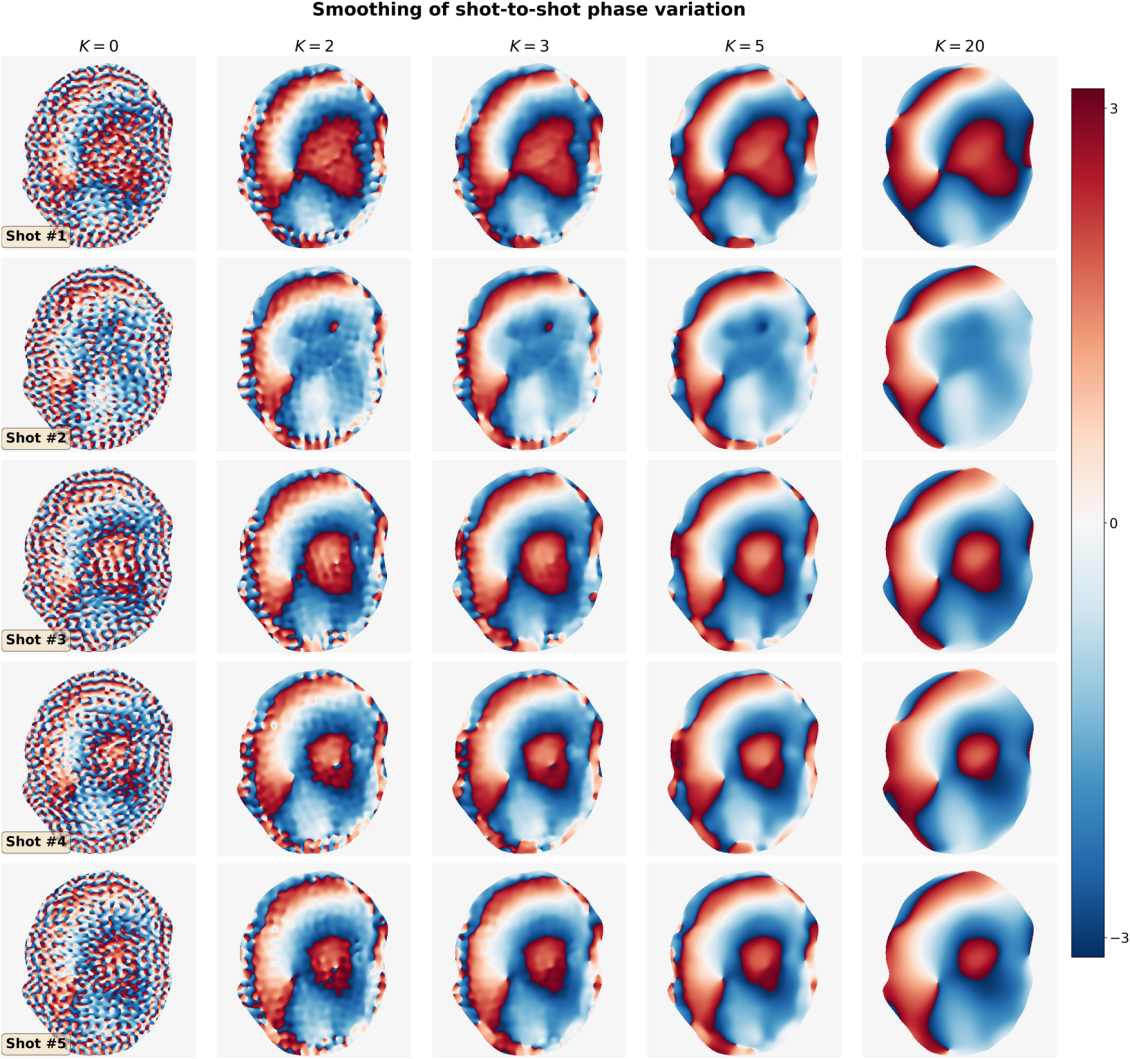
Smoothing of shot-to-shot phase variation according to Eq. (6). Navigators from Protocol #3 were reconstructed based on Step I in Section 2.5 and then used as the input (the column with K=0).

Navigators were acquired with the acceleration rate as listed in Table 1. Besides, the base resolution of navigators (e.g., 32 in Protocol #3 in Table 1) was smaller than imaging echoes. As a result, reconstructed navigator phases (refer to the first column in Fig. 3) from Step I in Section 2.5 are not spatially smooth. Such phases, when used in the shot-combined reconstruction, result in signal void artifacts in DW images. To address this problem, we utilized the phase smoothing procedure. As shown in Figure 3, the ripple-like phase artifact disappears at K=5, while retaining the shot-to-shot phase variation. In contrast, a larger K (e.g., K=20) makes the filter too strong and partially removes phase variation.

### 3.2 Comparison to MUSE and JULEP with four-shot iEPI acquisition

**Fig. 4.**
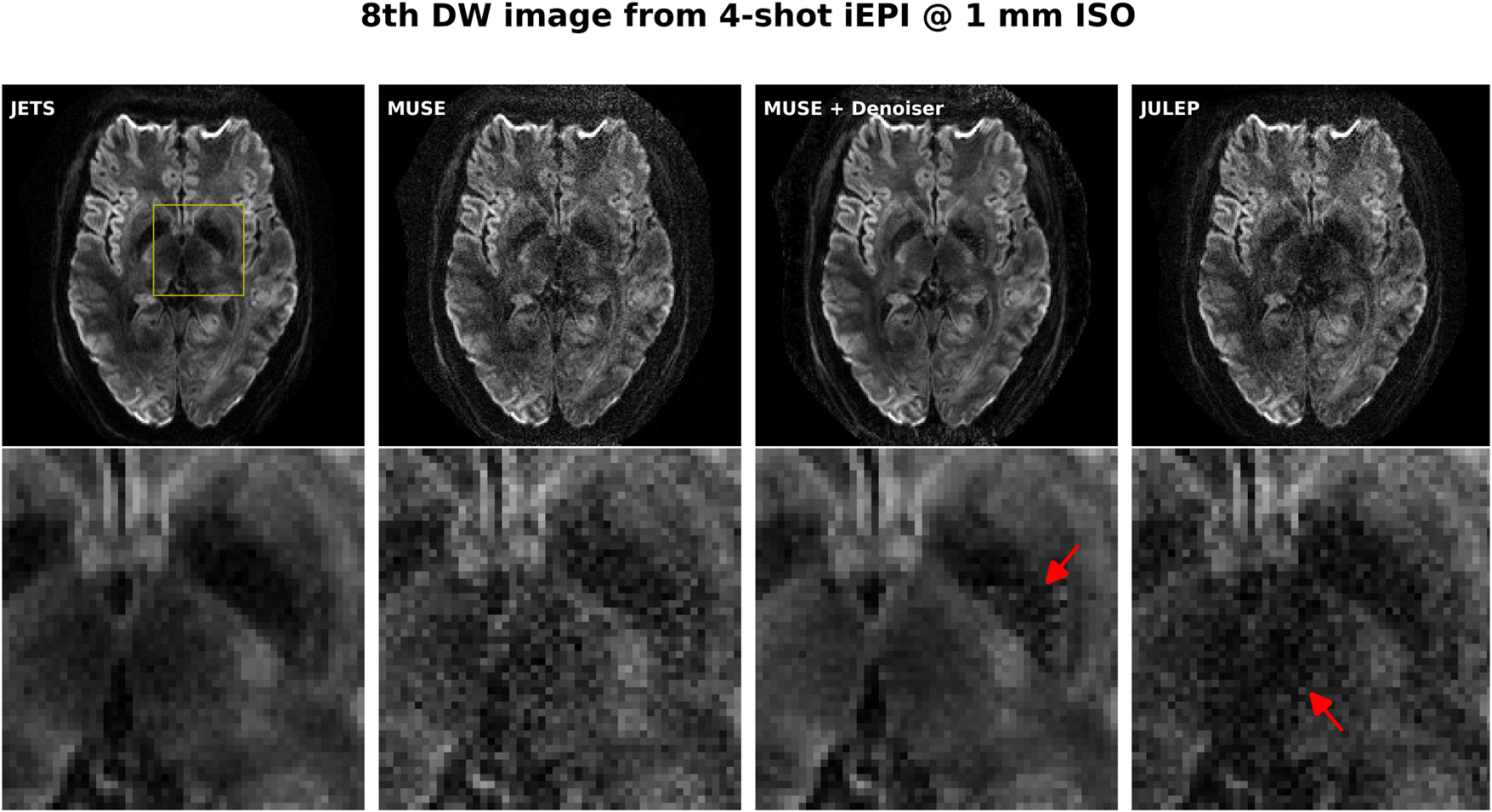
Reconstructed DW images (the 8th diffusion encoding) based on four-shot iEPI acquisition with 1 mm isotropic resolution (Protocol #1 in Table 1). Four reconstruction methods are compared (from left to right): JETS, MUSE, MUSE with denoiser, and JULEP. The 2nd row displays the magnified views of the yellow square. The image from the denoiser (3rd column) shows residual noise patterns within the globus pallidus (indicated by the red arrow). The JULEP reconstruction (4th column) shows signal dropout in the central region (indicated by the red arrow).

The iterative phase smoothing was also applicable to MUSE-type self-navigating reconstruction, where shot phases were reconstructed from imaging echoes. Figure 4 compares our proposed JETS with MUSE (Chen et al., 2013), MUSE with complex-valued local-PCA denoiser (Cordero-Grande et al., 2019), and JULEP (Dai et al., 2023). The residual noise from MUSE can be largely removed by the denoiser. However, when compared to JETS, the denoiser shows residual noise patterns within the globus pallidus (indicated by the red arrow). JETS also shows better denoising than JULEP. The reason is that JETS enforces spatial-diffusion regularization, whereas JULEP formulates structured low-rank regularization of the four shots for one diffusion encoding.

### 3.3 Retrospectively undersampling from the four-shot iEPI acquisition

**Fig. 5.**
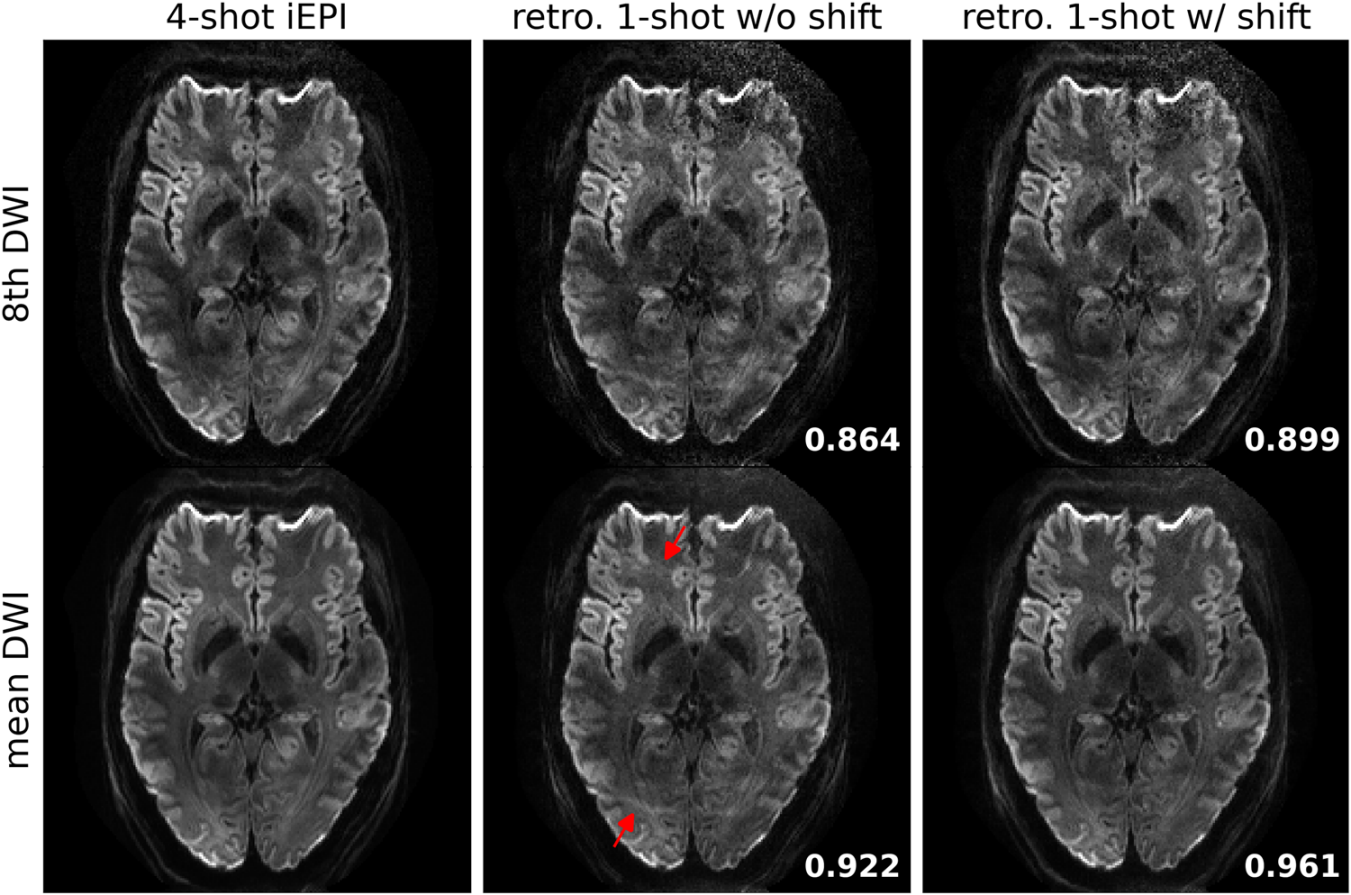
Quantitative validation of the proposed $k_{y}$-shift enoding sampling pattern based on four-shot iEPI acquisition with 1 mm isotropic resolution (Protocol #1 in Table 1). (Top) the 8th diffusion encoding and (bottom) mean DWI over 20 diffusion encodings. (1st column) JETS reconstruction of four-shot iEPI acquisition is used as the ground truth. The 2nd and the 3rd column displays JETS reconstruction of retrospectively undersampled one-shot acquisition without and with $k_{y}$ shifting, respectively. Residual aliasing artifacts are visible in the reconstruction without $k_{y}$ shifting, as indicated by the red arrows. Structural similarity (SSIM) values are computed and displayed in the bottom right corners.

JETS reconstruction results on the four-shot prospectively fully-sampled data from Protocol #1 in Table 1, as well as on the retrospectively undersampled one-shot data without and with the proposed $k_{y}$ shift are displayed in Figure 5. Residual aliasing artifacts are visible in the reconstruction without $k_{y}$ shifting, as indicated by the red arrows. In contrast, the $k_{y}$ shifting scheme supplies a complementary k- q-space sampling pattern, which is beneficial for joint reconstructions such as JETS. As shown in Figure 5, JETS results in improved SSIM values and reduced aliasing artifacts, when compared to the reconstruction without $k_{y}$ shifting. Figures 4 and 5 show a slice containing the globus pallidus with strong $T_{2}$-weighted contrast and highlighting the advantage of $k_{y}$-shift encoding in reducing undersampling artifacts.

### 3.4 Analysis of reconstruction parameters

**Fig. 6.**
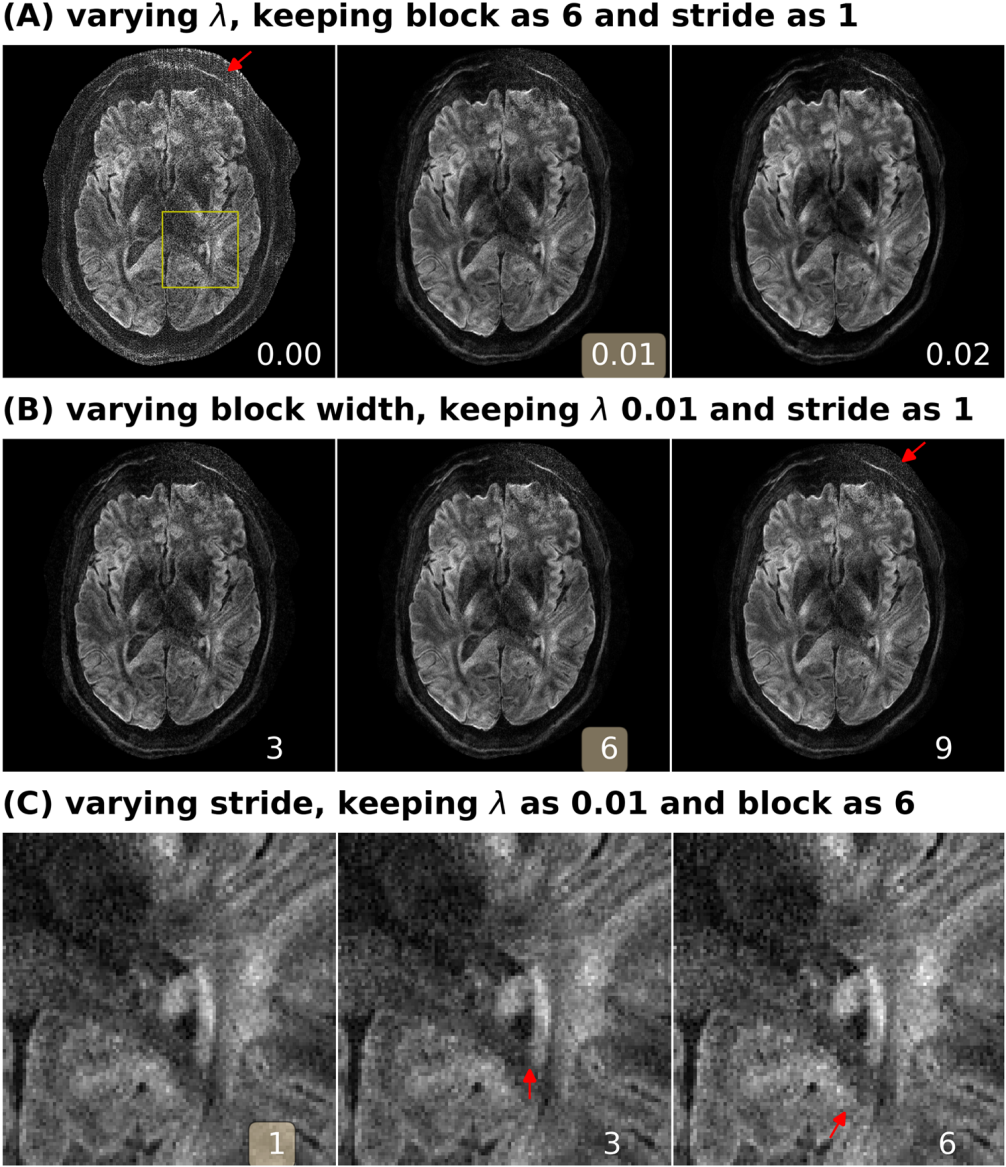
Analysis of reconstruction parameters based on the 3-scan trace acquisition with $0.5\times 0.5\times 2.0{\text{ mm}}^{3}$ (Protocol #3 in Table 1). Displayed are JETS reconstructed single-direction DW images. (A) Varying the regularization strength λ from 0 to 0.01 and 0.02. (B) Varying the block width from 3 to 6 and 9. The red arrow indicates increased noise with the large block width. (C) Varying the stride size from 1 to 3 (partially overlapping) and 6 (non-overlapping). The red arrows indicate blocky artifacts.

Here, we provide a systematic analysis of the proposed JETS reconstruction with LLR regularization applied to the spatial-diffusion dimension, as shown in Figure 6.

First, we varied the regularization strength λ. We tested values of 0, 0.01, and 0.02. The reconstruction with λ=0 in Eq. (7) corresponds to parallel imaging reconstruction without LLR regularization. It is worth noting that the proposed NAViEPI sequence demonstrates high-quality sub-millimeter DW images ($0.5\times 0.5\times 2.0mm^{3}$ in this example). The DW images can be further improved with the use of LLR regularization, that is, reduced noise, as seen in the reconstruction with λ= 0.01. Increasing λ (e.g., 0.02) further reduces noise, but at the cost of increased blurring. Therefore, λ= 0.01 was selected in this work.

Second, besides the regularization strength, we varied the block width. Reconstruction results for the data from Protocols #3 and #2 in Table 1 are displayed in Figure 6 (B) and SI Figure S6, respectively. With the above-mentioned normalization strategy, the reconstruction results show similar denoising effects. However, small block width (i.e., 3) suffers from residual blurring artifacts, as shown in SI Figure S6. Therefore, the block width of 6 was selected in this work.

Third, we varied the stride, that is, the step from one local patch to the next. The use of overlapping LLR (Fig. 6 (C) left) better suppresses blocky artifacts, compared to the partially overlapping (stride < block) LLR (Fig. 6 (C) middle) and the non-overlapping (stride = block) LLR (Fig. 6 (C) right).

### 3.5 Sampling efficiency of NAViEPI

**Fig. 7.**
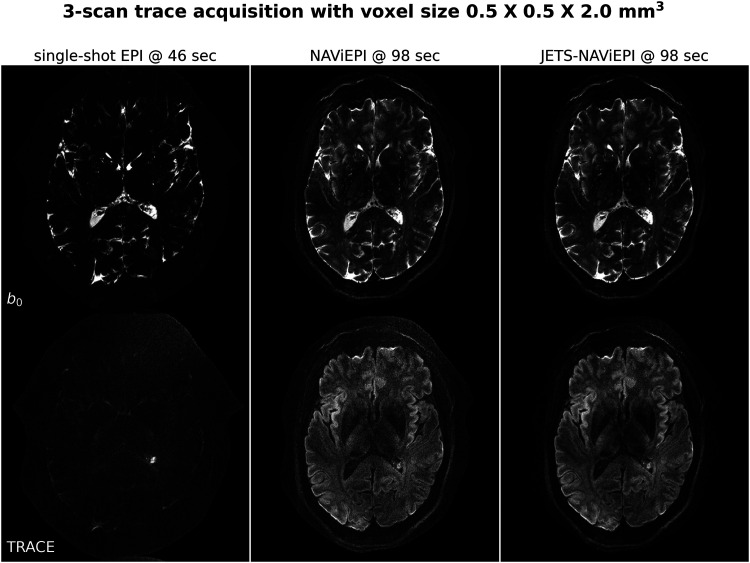
Sampling efficiency of the proposed NAViEPI sequence. Five-shot NAViEPI acquisition with the voxel size $0.5\times 0.5\times 2.0{\text{ mm}}^{3}$ (Protocol #3) was compared with single-shot EPI acquisition (Protocol #4). Both the 1st and the 2nd columns were reconstructed via parallel imaging without LLR regularization, whereas the 3rd column was reconstructed via JETS.

As shown in Figure 7, NAViEPI achieves sub-millimeter resolution (voxel size $0.5\times 0.5\times 2.0\;{\text{mm}}^{3}$) with the use of a five-shot acquisition. When compared to a single-shot acquisition with the same voxel size, the acquisition time of NAViEPI is about two times longer, but the image quality of NAViEPI is remarkably improved.

In the sub-millimeter imaging scenario, the increased base resolution requires longer TE (143 ms) in the single-shot acquisition, which results in significant signal loss due to $T_{2}$ relaxation. Therefore, sub-millimeter DWI necessitates multi-shot acquisition, which is subject to shot-to-shot phase variation and long scan time. However, NAViEPI solves both challenges. The five-shot acquisition reduces TE to 58 ms, and thus retains SNR significantly compared to the single-shot acquisition. Moreover, the JETS reconstruction can help to reduce noise and improve structural visibility.

**Fig. 8.**
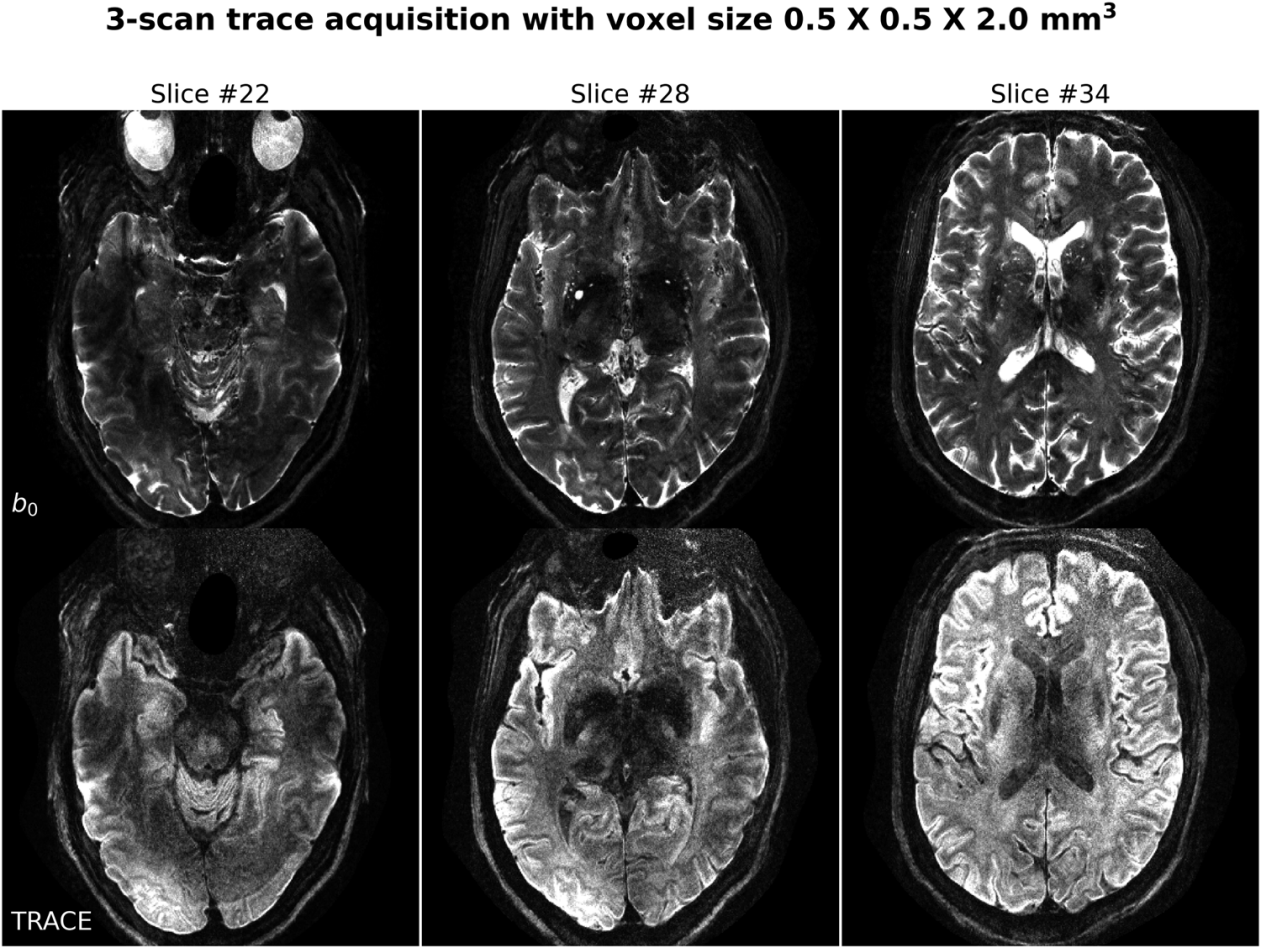
Reconstruction of the 3-scan trace acquisition with the voxel size $0.5\times 0.5\times 2.0{\text{ mm}}^{3}$ (Protocol #3) at different slices.

Figure 8 shows the JETS reconstructed $b_{0}$ and TRACE images in different slice locations. Admittedly, the lower brain region (e.g., slice #22) exhibits inhomogeneous and lower signal intensity than the upper slices. Such inhomogeneity can be alleviated with the use of multi-channel parallel transmission (Grissom et al., 2010; Katscher et al., 2003).

Here, Figures 6 and 7 show a slice with a benign lesion (the circular bright spot) within the left ventricle. Figure 8 displays three representative slices: (left) an inferior brain region with marked $B_{1}^{+}$ field inhomogeneity, (middle) the middle brain slice which shows susceptibility artifacts in the frontal region, and (right) a superior brain slice which shows the ventricle.

### 3.6 Diffusion tensor imaging

**Fig. 9.**
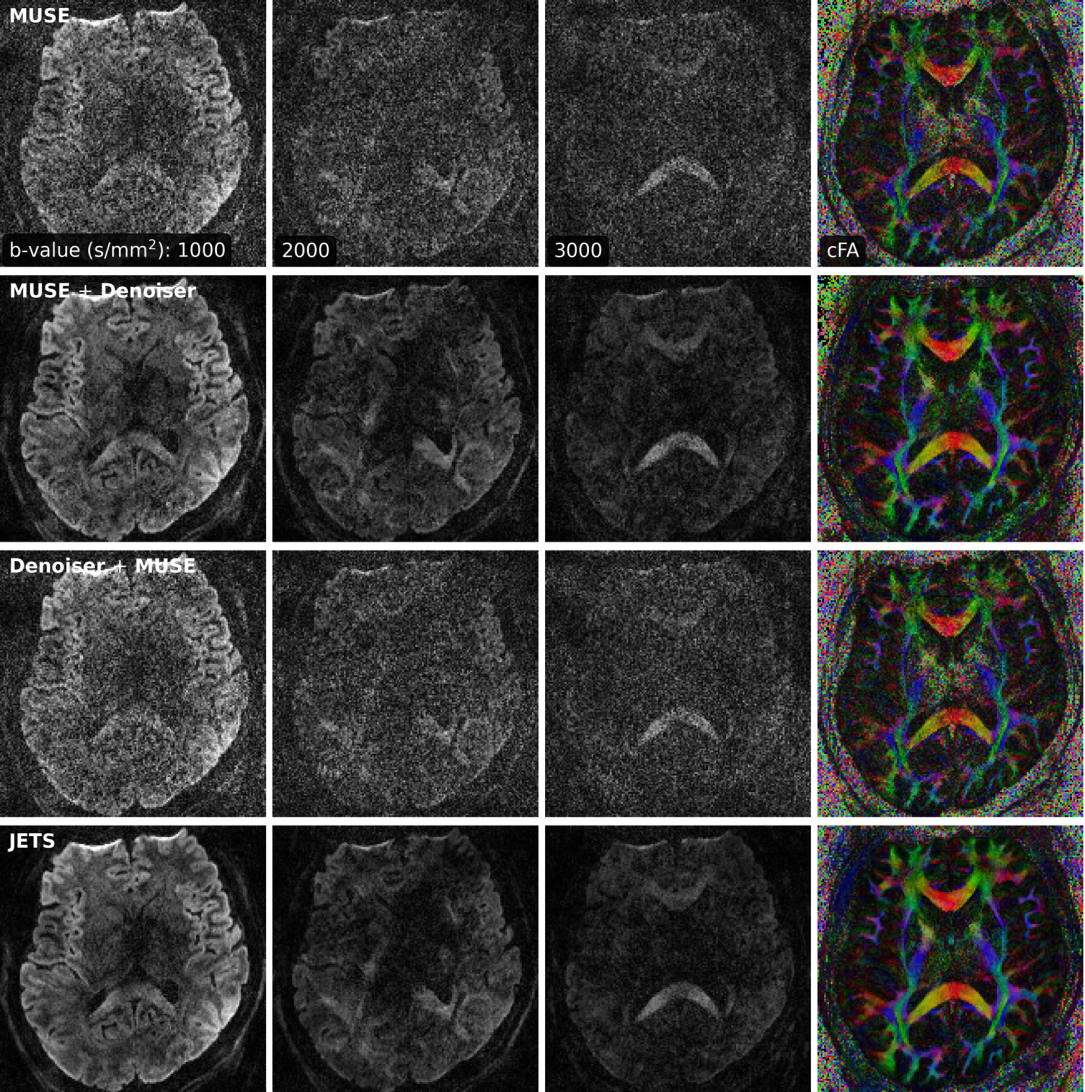
Comparison of three-shell DWIs and cFA maps with data acquired by Protocol #2 in Table 1. Reconstruction methods from top to bottom were MUSE, MUSE with the local-PCA denoiser, the application of the denoiser on shot images before the shot combination in MUSE, and the proposed JETS method.

Protocol #2 in Table 1 yields an acceleration factor of 6×3 per shot, resulting in severe noise amplification in MUSE reconstructed DWIs, as shown in Figure 9. Here, a slice that highlights the corpus callosum is displayed, and the diffusion direction at the b-value of 3000 s/mm^2^ with bright signal within the corpus callosum is shown. The local-PCA denoiser substantially removes noise, but the DWI at high b-values still illustrates more noise, compared to the proposed JETS reconstruction. On the other hand, we applied the local-PCA denoiser before the shot combination in MUSE. As shown in Figure 9, this approach is less effective compared to the application of the denoiser after the shot combination, because shot images were reconstructed from the central k-space region and have a coarse resolution.

## 4 Discussion

This work reports a novel DW-MRI technique, JETS-NAViEPI. NAViEPI (1) achieves the fast and efficient acquisition of both imaging and navigator echoes, (2) enforces consistent effective ESP between the two echoes, and (3) allows for undersampled iEPI as well as a large number of shots. Moreover, compared to the single-shot acquisition, joint k- q-slice reconstruction with $k_{y}$-shift encoding on NAViEPI retains SNR and reduces aliasing artifacts in DW images. As a result, JETS-NAViEPI renders high spatiotemporal resolution diffusion MRI protocols in 7 T, for example, a 3-scan trace acquisition with the voxel size $0.5\times 0.5\times 2.0{\text{ mm}}^{3}$ at 1.5 min.

One limitation of JETS-NAViEPI is the long reconstruction time due to the simultaneous reconstruction of all DW images and the use of overlapping locally low-rank regularization. The reconstruction for the Protocol #3 in Table 1 on an A100 GPU takes about 2 per multi-band slice. To reduce the computation time, coil compression algorithms (Buehrer et al., 2007; Huang et al., 2008) can be employed to reduce the number of coils for image reconstruction. Moreover, one can deploy multi-GPU distributed computing or modern optimization algorithms (e.g., stochastic gradient descent) (Ong et al., 2020) to speed up the reconstruction.

Neither the signal modeling in Eqs. (2) and (4) nor the LLR regularization considers the subject motion. In the presence of motion, the regularized reconstruction can degrade. To overcome this problem, scout-informed motion estimation and reconstruction (Polak et al., 2022) could be integrated into the framework.

Another potential extension of this work is to incorporate distortion correction. The standard distortion correction method is known as TOPUP (Andersson et al., 2003), which acquires two scans with opposing phase-encoding directions to obtain the field inhomogeneity map and then performs conjugate phase reconstruction to correct for distortion. Alternatively, a multi-echo acquisition could be used for the coil sensitivity reference scan, such that both coil sensitivity and $B_{0}$ field inhomogeneity maps could be reconstructed from the data.

This work employed a single regularization weight λ to enforce low rankness along the spatial-diffusion direction. However, SNR may be heterogeneous within the FOV. Therefore, one single regularization scalar may be inadequate to cover the whole FOV. Beyond this SVT-based reconstruction, one can seek to use machine learning to learn a q-space prior as the regularizer (Hammernik et al., 2018; Lam et al., 2019; Mani et al., 2021).

Although NAViEPI employs navigators for the acquisition of shot-to-shot phase variation, it is worth noting that phase behavior depends on several hard-to-control factors such as pulsatile motion, bulk motion, locations within the brain, and diffusion sensitization strength. Therefore, more comprehensive modeling or post-processing such as image registration can be considered in future work.

This work compared LLR regularized JETS to MUSE post-processed by the local PCA denoiser (Cordero-Grande et al., 2019). Both the LLR regularization and the local PCA denoiser are based on the principle that low rankness exists in the spatial-diffusion dimension (Moeller et al., 2021), where the spatial content is extracted from local patches within the full image volume and the diffusion dimension is from the q-space encoding. One could integrate the automatic noise estimation based on the Marchenko-Pastur law for the determination of the thresholds in the LLR regularization to synergize these two methods.

While this work reconstructs all DW images and then performs model fitting, an alternative approach is to directly estimate $b_{0}$ and diffusion tensors from measured k- q-space data using model-based reconstruction (Dong et al., 2018; Knoll et al., 2015; Shafieizargar et al., 2023). Compared to DW image reconstruction, model-based reconstruction solves for a fewer number of unknowns, but requires strict diffusion tensor modeling and the use of nonlinear least square solvers.

## 5 Conclusions

We demonstrated the JETS-NAViEPI technique, which integrates a $k_{y}$-shifted encoding navigator-based interleaved EPI sequence and joint reconstruction with overlapping locally low-rank regularization for high spatial-angular-temporal resolution DW-MRI at 7 T. This technique allows for high-quality DW image reconstruction with accelerated acquisitions.

## Supplementary Material

Supplementary Material

## Data Availability

In the spirit of reproducible and open science, we publish our source code (https://github.com/ZhengguoTan/sigpy) as well as the raw k-space data (https://doi.org/10.5281/zenodo.10474402). We also provide interactive demonstrations of the reconstruction procedure (https://github.com/ZhengguoTan/NAViEPI).
