# Supplementary material for: Accelerated diffusion-weighted magnetic resonance imaging at 7 T: Joint reconstruction for shift-encoded navigator-based interleaved echo planar imaging (JETS-NAViEPI)
Source: Imaging Neurosci (Camb). 2024 Feb 5;2:imag-2-00085. doi: 10.1162/imag_a_00085 (PMC12235561; doi:10.1162/imag_a_00085)
Supplement: Supplementary Material [file imag_a_00085-supp.pdf]

Supplementary Information

Accelerated Diffusion Weighted Magnetic Resonance Imaging at 7 T: Joint Reconstruction for Shift-Encoded Navigator-based Interleaved Echo Planar Imaging (JETS-NAViEPI)

Zhengguo Tan, Patrick A. Liebig, Robin M. Heidemann, Frederik B. Laun, Florian Knoll

Here we aim to reproduce the results. Another subject with informed consent was recruited and measured by all protocols listed in Table 1 in the main manuscript.

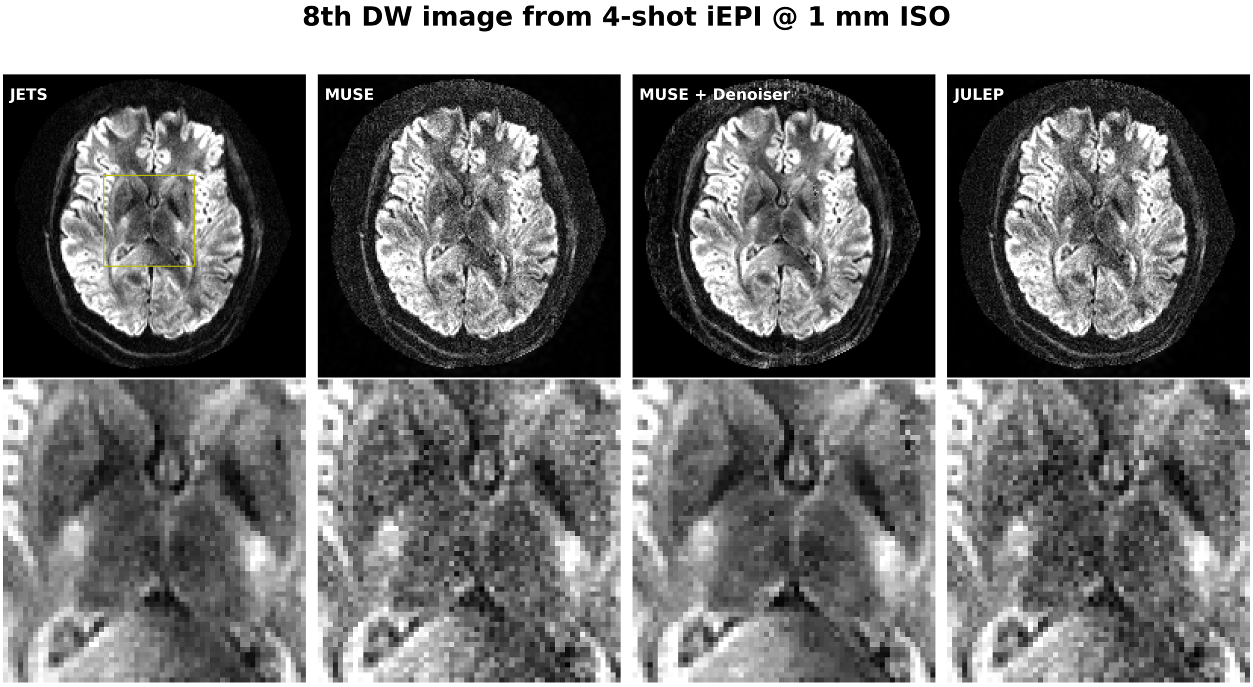

SI Figure S1: Reproducing Protocol #1. Reconstructed DW images (the 8th diffusion encoding) based on 4-shot iEPI acquisition with 1 mm isotropic resolution. Four reconstruction methods are compared (from left to right): JETS, MUSE, MUSE with denoiser, and JULEP. The 2nd row displays the magnified views of the yellow square.

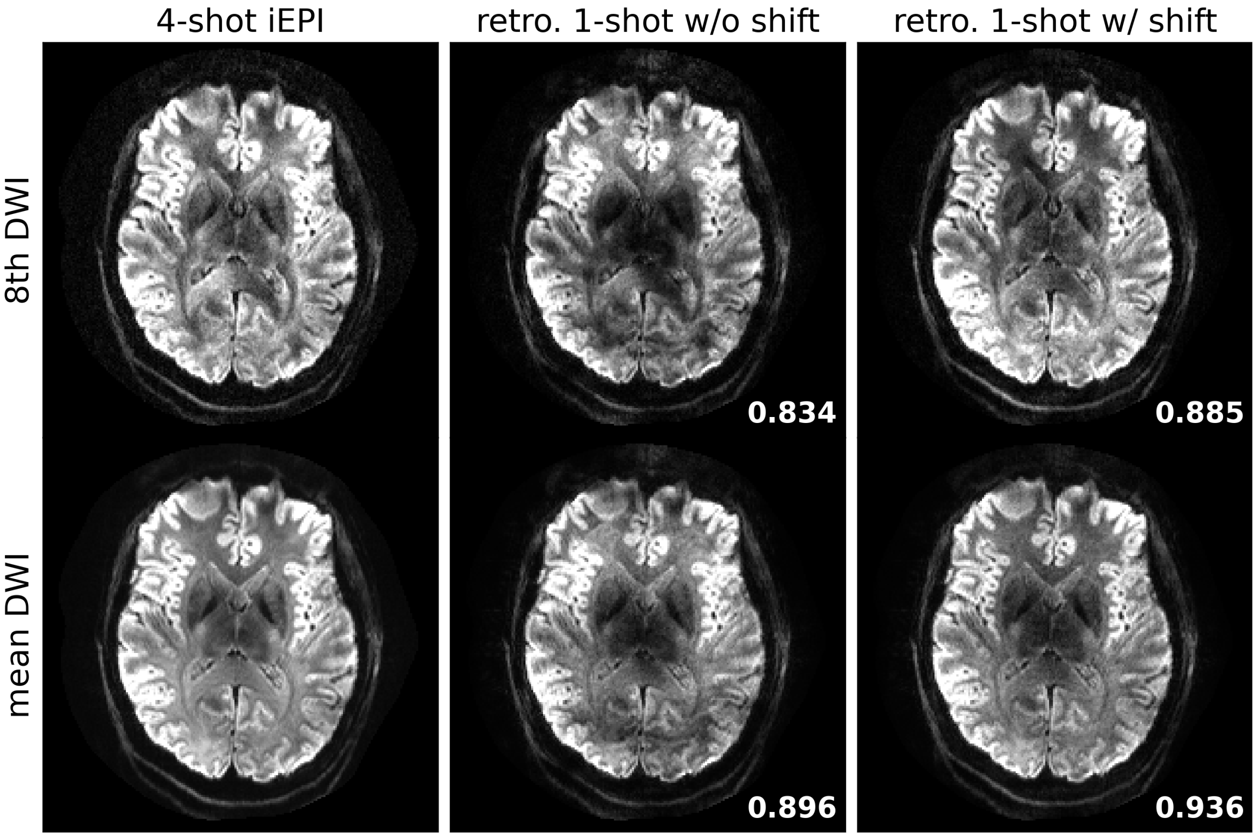

SI Figure S2: Reproducing Protocol #1. Quantitative validation of the proposed k_y-shift enoding sampling pattern based on 4-shot iEPI acquisition with 1 mm isotropic resolution. (Top) the 8th diffusion encoding and (bottom) mean DWI over 20 diffusion encodings. (1st column) JETS reconstruction of 4-shot iEPI acquisition is used as the ground truth. The 2nd and the 3rd column displays JETS reconstruction of retrospectively undersampled 1-shot acquisition without and with k_y shifting, respectively.

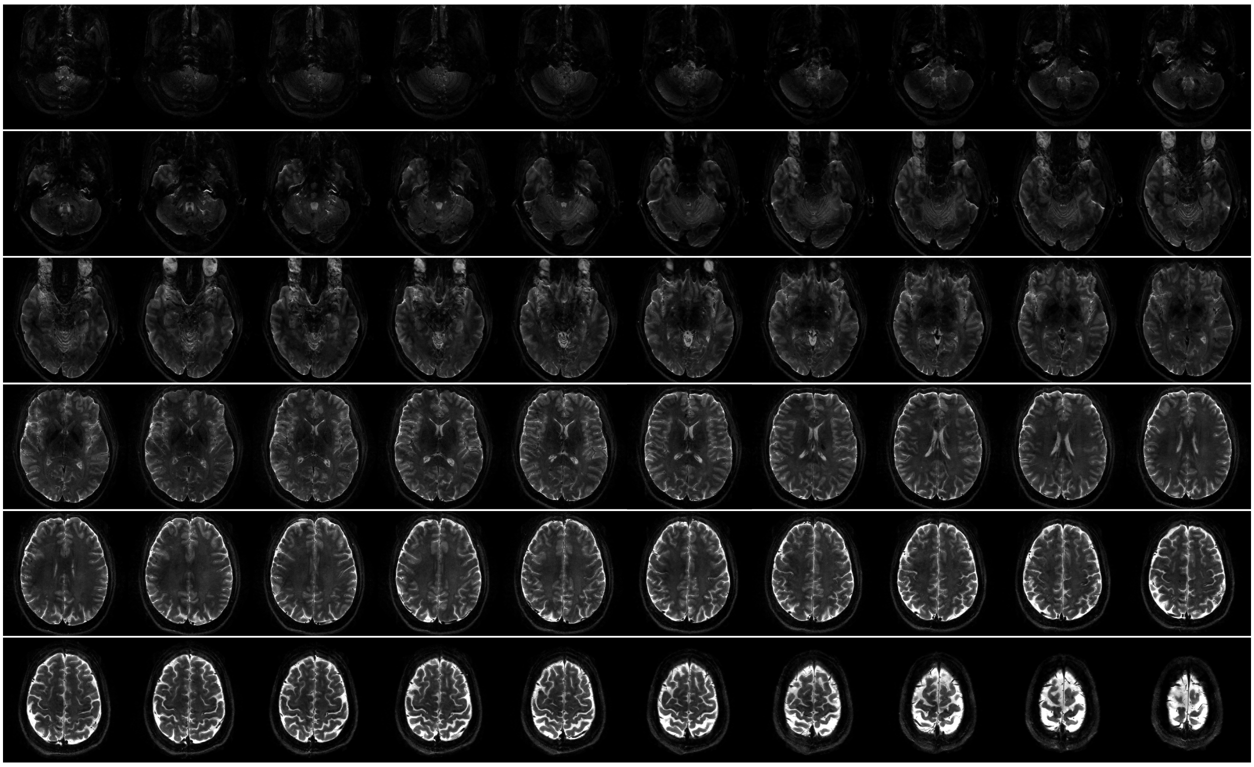

SI Figure S3: Reproducing Protocol #3. Reconstructed b_0 images from the 3-scan trace acquisition with the voxel size $0.5 \times 0.5 \times 2.0$ mm^3.

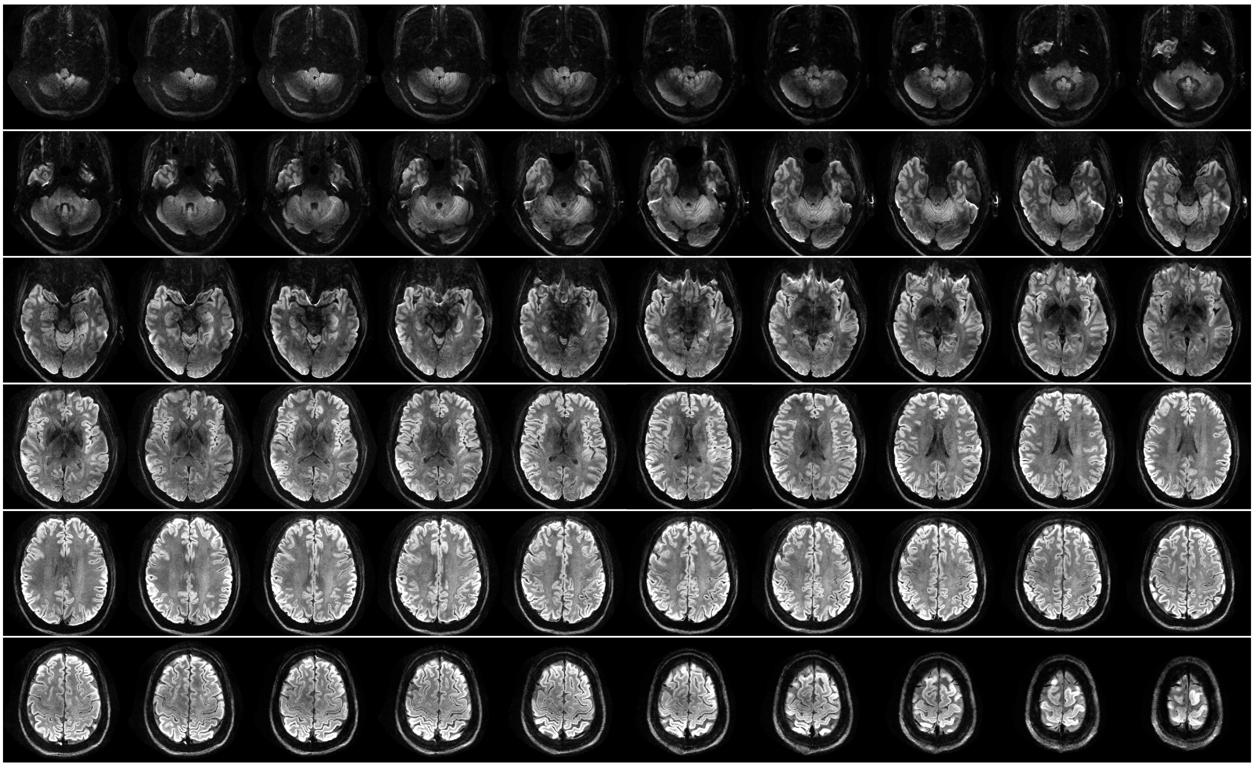

SI Figure S4: Reproducing Protocol #3. Reconstructed TRACE images from the 3-scan trace acquisition with the voxel size $0.5 \times 0.5 \times 2.0$ mm^3.

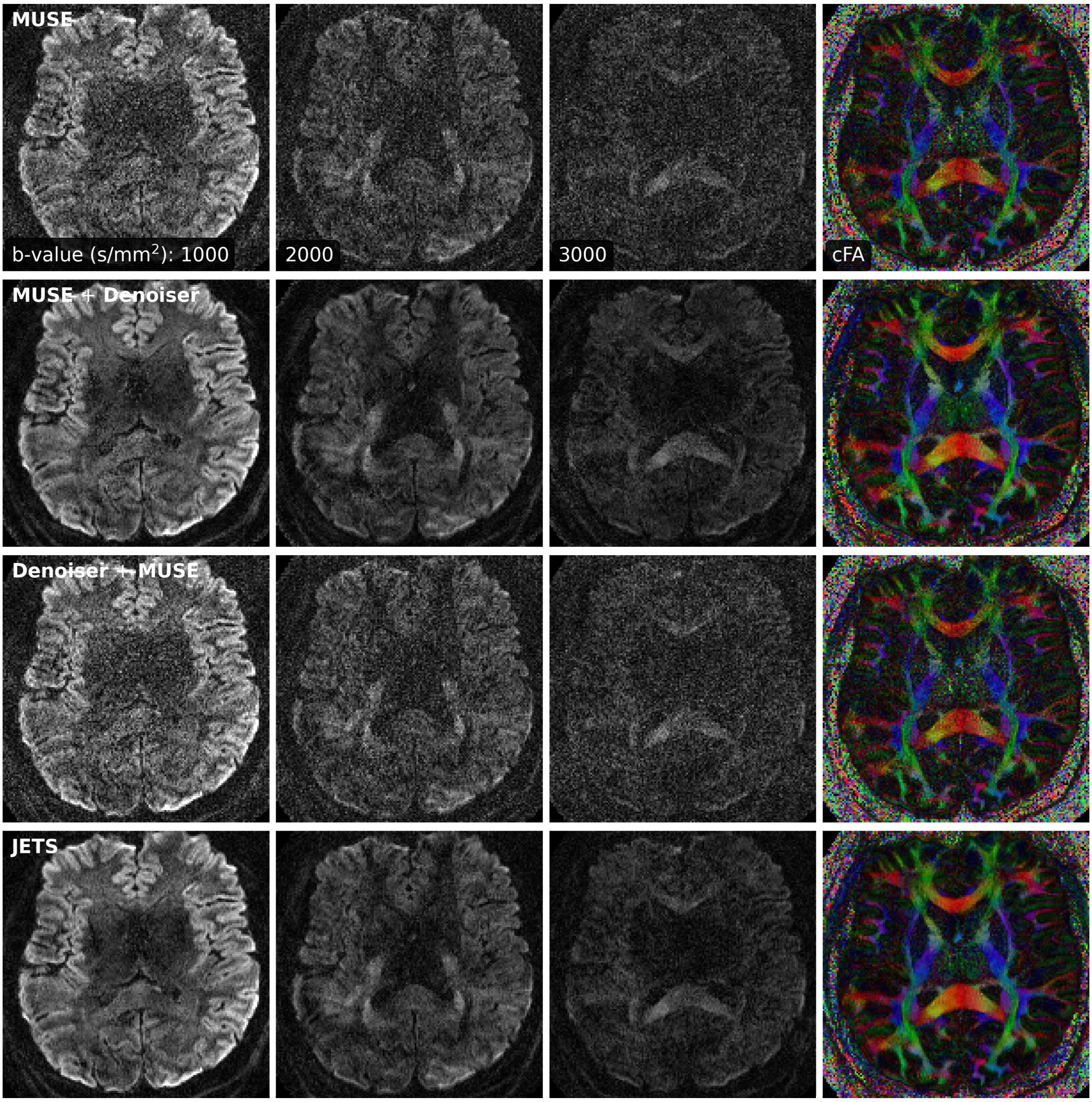

SI Figure S5: Reproducing Protocol #2. The FOV and bandwidth were adapted as $200\,\mathrm{mm}$ and 1086 Hz/pixel, respectively. Comparison of three-shell DWIs and cFA maps reconstructed by (top to bottom) MUSE, MUSE with the local-PCA denoiser, MUSE with the local-PCA denoiser applied before the multi-shot combination, and the proposed JETS method, respectively. The local-PCA denoiser, when applied to shot images (3rd row), is less effective compared to its application to shot-combined images (2nd row). The reason is that shot images are reconstructed from the central k-space data, and thus have coarse resolution.

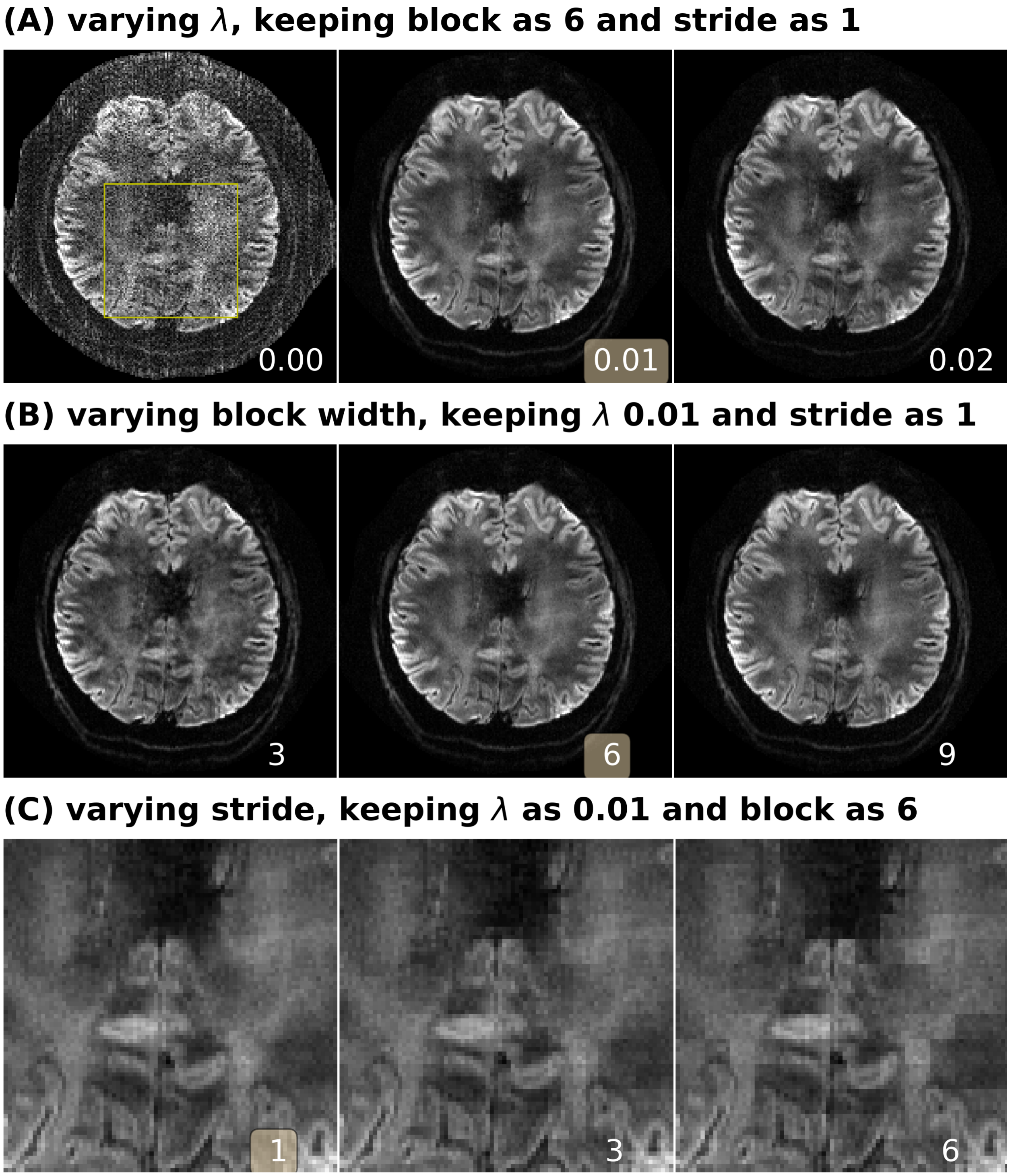

SI Figure S6: Analysis of reconstruction parameters based on the 3-shell acquisition with 1 mm^3 isotropic resolution (Protocol #2 in Table 1). Displayed are JETS reconstructed single-direction DW images. **(A)** Varying the regularization strength λ from 0 to 0.01 and 0.02. **(B)** Varying the block width from 3 to 6 and 9. The red arrow indicates increased noise with the large block width. **(C)** Varying the stride size from 1 to 3 (partially overlapping) and 6 (non-overlapping). The red arrows indicate blocky artifacts.
